# Achieving sustainable medical tourism: unpacking privacy concerns through a tripartite game theoretic lens

**DOI:** 10.3389/fpubh.2024.1347231

**Published:** 2024-04-09

**Authors:** Ran Wang, Songtao Geng

**Affiliations:** ^1^College of International Tourism and Public Administration, Hainan University, Haikou, China; ^2^Faculty of History and Tourism Culture, Inner Mongolia Minzu University, Tongliao, China

**Keywords:** medical tourism, privacy protection, evolutionary game theory, incentives and penalties, sustainable growth

## Abstract

**Introduction:**

Medical tourism has grown significantly, raising critical concerns about the privacy of medical tourists. This study investigates privacy issues in medical tourism from a game theoretic perspective, focusing on how stakeholders’ strategies impact privacy protection.

**Methods:**

We employed an evolutionary game model to explore the interactions between medical institutions, medical tourists, and government departments. The model identifies stable strategies that stakeholders may adopt to protect the privacy of medical tourists.

**Results:**

Two primary stable strategies were identified, with E_6_(1,0,1) emerging as the optimal strategy. This strategy involves active protection measures by medical institutions, the decision by tourists to forgo accountability, and strict supervision by government departments. The evolution of the system’s strategy is significantly influenced by the government’s penalty intensity, subsidies, incentives, and the compensatory measures of medical institutions.

**Discussion:**

The findings suggest that medical institutions are quick to make decisions favoring privacy protection, while medical tourists tend to follow learning and conformity. Government strategy remains consistent, with increased subsidies and penalties encouraging medical institutions towards proactive privacy protection strategies. We recommend policies to enhance privacy protection in medical tourism, contributing to the industry’s sustainable growth.

## 1 Introduction

Medical tourism has evolved into a global phenomenon, characterized by patients seeking medical procedures across borders, motivated by factors such as cost-efficiency, timely care, and access to specialized services (1–3). According to Market.us Media, the industry is expanding at a rate of 15–25% annually, with countries like India, Thailand, and Singapore leading as preferred destinations due to their combination of advanced healthcare facilities and competitive pricing (4). However, the burgeoning growth raises questions about the sustainability of such practices in the face of increasing demand for cross-border medical services and the corresponding need for international healthcare policy harmonization (5).

Privacy in the medical tourism industry is not merely a legal requirement but also a fundamental patient right and a critical component of quality care (6). Confidentiality concerns are heightened in this context due to diverse cultural expectations, legal systems, and potential language barriers that can result in misunderstandings and breaches of privacy (7). Ensuring the security of sensitive personal health information becomes paramount as it can impact patient well-being and trust in the healthcare system (8).

Protecting the privacy of medical tourists is fraught with challenges that range from regulatory disparities to technological vulnerabilities (9). Variances in national laws regarding data protection can lead to inconsistencies in personal health information handling and increase the complexity of legal recourse for patients in the event of a privacy breach (10). The inter-jurisdictional transfer of health data often lacks a unified legal framework to guide practice and policy (11). Furthermore, the rise of digital health records and telemedicine consultations necessitates stringent cybersecurity protocols to mitigate risks such as unauthorized access and data theft (12). Such complexities underscore the need for an international consensus on privacy standards in medical tourism.

Despite burgeoning literature on medical tourism, significant gaps remain in understanding how privacy protection is operationalized across different jurisdictions. While some nations have robust data protection laws, others offer little to no protection for medical tourists, leading to an uneven patchwork of regulations that do not adequately address the transnational nature of medical tourism (13, 14). Further research is needed to identify specific weaknesses in existing privacy protection frameworks and to propose solutions that can work across borders.

Privacy breaches in medical tourism can have severe consequences for both patients and service providers. For patients, breaches can lead to discrimination, financial loss, and emotional distress, while for service providers, they can result in reputational damage, loss of business, and legal liabilities (15, 16). The global nature of medical tourism calls for a strategic approach to privacy protection that is multi-faceted and international in scope. Existing strategies often focus on national regulations, which are insufficient given the cross-border flow of health information (17). A comprehensive strategy must include international legal frameworks, standardized protocols for data security, and cooperation between destination and source countries to ensure that the rights of medical tourists are safeguarded (18, 19). Only through such a strategic approach can privacy risks be mitigated and the sustainable growth of the medical tourism industry be supported.

In summation, concerning the realm of privacy protection within medical tourism, existing literature, though progressive, presents certain persisting quandaries and challenges. Specifically: (1) How can an evolutionary game-theoretic model be constructed to incorporate interactions among government departments, medical institutions, and medical tourists? (2) What delineates the optimal evolutionary strategies among pivotal stakeholders? (3) What characteristics define the decision-making processes of these evolutionary game participants? (4) Which determinants, when modulated, expedite the attainment of the optimal evolutionary stability strategy?

This study highlights significant advancements in understanding the dynamic nature of privacy strategies in medical tourism, offering benefits in theory, industry practice, and policy-making. Theoretically, it moves beyond static models, providing a more intricate view of real-world healthcare interactions and improving the predictive capabilities of these models. Practically, it presents strategies that can help medical tourism providers gain a competitive advantage through enhanced trust and patient loyalty, emphasizing that effective privacy management is key to quality care and the industry’s sustainable growth. Finally, the study forms a base for policy development, suggesting a flexible framework informed by evolutionary game theory, promoting international cooperation, and positioning privacy as crucial in medical tourism. Governments are encouraged to support institutions following these privacy norms, fostering innovation while ensuring industry growth and protecting patient privacy.

## 2 Literature review

### 2.1 Origin and background of privacy issues in medical tourism

The convergence of medical tourism and technological advancements has reshaped the healthcare landscape, introducing both transformative benefits and novel challenges (20). The adoption of information technology (IT) in the medical field has revolutionized patient care, offering streamlined operations, real-time communication, and enhanced diagnosis and treatment (21). Electronic health records (EHRs), telemedicine, and mobile health apps have brought unprecedented convenience and efficiency (22, 23). However, the digital nature of these tools also introduces vulnerabilities. Unauthorized access, data breaches, and cybersecurity threats pose significant risks to the confidentiality and integrity of patient information, especially when data crosses borders in medical tourism scenarios (24).

Medical tourism’s rise has been inextricably tied to globalization, characterized by the free flow of people, information, and technology across borders (25). While this interconnectivity has facilitated access to world-class healthcare, it has also exposed medical tourists to heterogeneous data protection standards. Several incidents, particularly in emerging medical tourism hubs, have spotlighted the repercussions of lax privacy protocols and diverse regulatory frameworks. During the period from 2015 to 2019, approximately 250 million individuals experienced breaches in healthcare privacy (16). Hackers and other IT incidents accounted for the largest proportion of healthcare data breaches reported in 2020. During the year, there were 429 reported incidents related to hacking/IT incidents, making up 66.82% of all breach events, with the number of records affected by these breaches reaching an even higher percentage of 91.99% (26). From inadvertent data leaks to orchestrated cyber-attacks (27, 28), medical tourists have become targets, underscoring the imperative of establishing robust, universally acknowledged privacy norms (29).

In summary, the emergence of medical tourism as a formidable sector in global healthcare is a double-edged sword. While it offers myriad opportunities for economic growth and global collaboration, it also surfaces challenges related to patient data privacy, cultural congruence, and equitable access to healthcare resources.

### 2.2 Behavioral patterns of medical tourists in privacy protection

Understanding the motivations and behaviors of medical tourists is pivotal when addressing privacy concerns, which stand out among the numerous factors influencing their decisions to seek treatment abroad (30). Privacy concerns are not just theoretical but have real-world implications, as evidenced by recent studies showing a trend of increased privacy awareness among medical tourists, a consequence of the digitization boom in healthcare (31). This vigilance is rooted in high-profile incidents of data breaches and the unauthorized sharing of sensitive health information, which have not only legal ramifications but also profound personal impacts, such as the threat of stigmatization or discrimination following the disclosure of certain health conditions (32).

The influence of cultural background on privacy concerns is notable and diverse (33). For example, Western patients often demand high levels of anonymity and stringent data protection, a stance likely influenced by a regulatory environment that emphasizes individual privacy rights. On the contrary, in certain Asian cultures, where communal decision-making is prevalent, there might be a more relaxed attitude toward data sharing within the extended family network (34). However, this cultural tendency does not diminish the necessity for privacy protection but rather emphasizes the need for culturally sensitive privacy protocols that cater to the expectations of medical tourists from different backgrounds (35).

Medical institutions must recognize and respond to these cultural subtleties by tailoring their privacy protocols. Incorporating evolutionary game theory, we can anticipate and model these varied behavioral patterns as strategies that evolve over time. Medical tourists adapt their privacy demands based on experiences and information about past privacy breaches, and medical providers, in turn, evolve their privacy protections to meet these expectations and to maintain their reputational standing in a competitive market. As such, the cultural and behavioral dimensions of privacy concerns become dynamic factors in the game-theoretical analysis of medical tourism, informing the evolution of privacy protection strategies and policy development.

### 2.3 Evolutionary game theory in privacy protection

Evolutionary game theory (EGT) offers a pertinent model for addressing privacy issues in medical tourism due to its ability to capture the dynamic nature of interactions among diverse stakeholders (36). Unlike classical game theory, which assumes perfect rationality, EGT assumes that players adapt their strategies over time based on their experiences (37). This is especially relevant in medical tourism, where providers and consumers continuously adjust their behavior in response to privacy concerns and policy changes.

In the realm of healthcare, privacy protection is a dynamic process involving multiple stakeholders—patients, healthcare providers, and policy-makers—who interact repeatedly. These interactions often resemble a complex adaptive system where strategies evolve based on past outcomes and anticipated future risks (38). EGT encapsulates this process by modeling how stakeholders may adapt their privacy strategies in response to the evolving digital landscape and its associated threats, such as data breaches (39, 40). For example, the shift toward digital health records has heightened the risk of privacy breaches. This has led healthcare providers to engage in an evolutionary “arms race,” constantly developing and adopting more advanced privacy protection measures to safeguard patient data and comply with tightening regulations (41).

In medical tourism, the temporary and overlapping relationships between medical tourists, healthcare providers, and regulators across borders create a complex network of interactions. EGT serves as a useful analytical tool to understand and predict how privacy norms and protection strategies may evolve in this context (42). For instance, as healthcare providers strive to attract international patients, they are motivated to enhance their privacy protections. In turn, patients seek out destinations that not only provide high-quality medical services but also ensure the confidentiality of their health information (43).

Evolutionary Game Theory (EGT) provides a useful framework for understanding privacy protection strategies in medical tourism, but it has limitations. It may not fully capture the impact of diverse cultural values and regulatory environments on stakeholders’ decisions. Therefore, empirical research, such as case studies and surveys, is essential to validate EGT’s effectiveness in real-world healthcare settings. Additionally, concrete policy recommendations are needed to apply EGT effectively in medical tourism. This involves integrating EGT insights into policy-making, considering cultural differences and international regulatory complexities. Bridging the gap between EGT’s theoretical aspects and practical applications in medical tourism is crucial. This can be achieved by adapting the model to the specific needs of the medical tourism industry and using real-life examples to guide its application in developing effective privacy strategies and regulations.

In conclusion, EGT provides a robust theoretical foundation for understanding and guiding the evolution of privacy protection strategies in medical tourism. Its application helps to predict how stakeholders might adapt their behaviors to ensure privacy, given the sector’s unique challenges. This analysis paves the way for more informed policy-making and a better understanding of the strategic considerations underlying privacy protection in the healthcare sector.

In sum, privacy concerns in medical tourism are multi-faceted, shaped by technological advancements, individual behaviors, and the dynamics among governments, medical institutions, and tourists. The challenge lies in striking a balance, ensuring that while medical tourism thrives, patient privacy remains uncompromised. This study presents an innovative examination of the mechanisms underlying medical tourism privacy protection through the formulation of a tripartite evolutionary game model. Such a model offers a comprehensive theoretical foundation for enhancing privacy measures in medical tourism, thereby ensuring its robust and efficient progression.

The salient contributions of this research are enumerated as follows:(1) The initiative to use a three-party evolutionary game theory stands as a novel approach in the academic discourse surrounding medical tourism, aiming to bring a deeper understanding of the privacy protection dynamics at play. (2) This research undertakes a rigorous exploration of the ramifications associated with privacy protection in medical tourism, emphasizing governmental punitive intensities, subsidy mechanisms, reward dynamics, and compensatory frameworks implemented by healthcare institutions. The insights procured from this study hold substantial pragmatic implications, proffering pivotal guidance for bolstering privacy safeguards within the medical tourism paradigm. (3) The study is positioned to offer rich insights that can potentially guide and influence policy formulations, thereby encouraging a safe and trustworthy environment for medical tourism.

## 3 Model construction

### 3.1 Problem description

In the evolving landscape of medical tourism, the preservation of privacy stands out as a paramount concern. The game relationship among medical tourists, medical institutions, and government departments presents a complex dynamic, underpinned by diverse interests, incentives, and potential outcomes.

Medical Tourists: Medical tourists are primarily driven by the need for quality, affordable healthcare coupled with the expectation of privacy concerning their medical records and personal data. Their decisions, shaped by trust, often pivot on perceived confidentiality assurances and past privacy preservation reputation of medical institutions and destination countries (44). Simultaneously, medical tourists form and adjust their cognitive and emotional impressions of medical tourism destinations based on various motivations and risk factors, thereby influencing their travel decisions and behaviors (45).

Medical Institutions: Due to the negative impact of risks associated with healthcare attributes on the image of a destination (46), healthcare providers in the medical tourism sector are eager to maintain a competitive advantage by making significant investments in privacy measures. They understand the crucial role these measures play in attracting and retaining international patients to avoid damage to the destination’s image caused by risks of privacy breaches in medical tourism. However, these institutions also face pressure to share data for research, marketing, and sometimes, with governmental bodies for regulatory reasons (47).

Government Departments: Governments play a dual role. On one hand, they are regulators, setting privacy standards and ensuring compliance. Their policies influence the privacy landscape, either bolstering trust through stringent regulations or diluting it by allowing data access and sharing for broader societal or economic reasons (48). On the other hand, governments are also promoters of medical tourism, where data might be used to enhance the sector’s competitive positioning.

The dynamic interplay among these stakeholders can be likened to a three-party game, where each party’s strategy is influenced not only by their individual payoffs but also by the actions and expected responses of the other players. This interdependence, often non-linear, is governed by trust, information asymmetry, and the changing privacy regulations landscape (49).

While this triad game relationship underscores the significance of collaborative strategy formulation, it also emphasizes the need for transparent communication, robust privacy protection mechanisms, and adaptive regulations that align with the rapidly evolving medical tourism sector’s demands.

### 3.2 Model assumptions

Based on the analysis of the interests and conflicts of the parties involved in the protection of privacy for medical tourists, three main stakeholder groups have been selected for the study: medical institutions, medical tourists, and government departments. The following hypotheses are made regarding the behavior of the three parties.

*Hypothesis 1*: The three parties involved in protecting the privacy of medical tourists are all boundedly rational and prioritize maximizing their own interests as the primary goal in the process.

*Hypothesis 2*: The strategy choices of medical institutions are (active protection, negative protection). When medical institutions fail to adequately protect the privacy of medical tourists, they may exploit the personal information of medical tourists for financial gain. Medical tourists’ strategic choices are (seek accountability, forgo accountability). When medical tourists discover that their privacy has been violated, they may choose to hold medical institutions accountable or not. However, medical tourists may also mistakenly blame the medical institution that diligently safeguards their privacy due to insufficient information and cognitive bias. The strategic choices of the government departments are (strict supervision, loose supervision). Under strict supervision, if medical tourists hold medical institutions accountable for privacy breaches, the institutions can be investigated and penalized. If medical institutions are not held accountable for privacy breaches, the probability of government departments being able to investigate and prosecute these institutions for a breach is p (0 < *p* < 1). Under loose supervision, the probability of successfully holding medical institutions accountable by medical tourists is m, (0 < m < 1). It is assumed that the probability of medical institutions actively safeguarding privacy is (1-x), the probability of passively safeguarding privacy is y, the probability of medical tourists holding medical institutions accountability for privacy breaches is x, and the probability of strict supervision is z. Correspondingly, (1-y) and (1-z) are the probabilities of forgoing accountability and loose supervision, respectively. The probabilities of x, y, z range [0, 1].

*Hypothesis 3*: The business revenue for medical institutions in the process of providing services to medical tourists is recorded as I. When selecting the “active protection” strategy, there will be certain costs involved in purchasing privacy protection equipment, network security hardware and software, as well as importing information protection technology and hiring professionals. These costs are recorded as C_1_. When government departments actively supervise, they will subsidize medical institutions that adopt the “active protection” strategy, which is recorded as S. When medical institutions passively protect privacy, the cost of protection is denoted as C_2_, and C_2_ < C_1_. Meanwhile, medical institutions stand to benefit ΔI from actions that disclose privacy, but they are also at risk of being held accountable by medical tourists and investigated by government departments. When medical institutions are held accountable and investigated for a privacy breach, the compensation to the medical tourists is recorded as C_0_, the government penalty is recorded as P_0_, and P_0_ ≤ ΔI. The image loss when a medical institution actively protects the privacy of medical tourists but is held accountable is recorded as L_1_, and the image loss caused by a medical institution being held accountable for leaks that occur in the context of negatively protecting the privacy is recorded as L_2_, and L_1_ < L_2_.

*Hypothesis 4*: Medical tourists are the beneficiaries of privacy protection. They engage in medical tourism at a cost (C_3_), in exchange for a valuable experience (V). When their privacy is compromised, it can result in emotional and economic losses, which will be recorded as L_3_. The cost of seeking accountability, in terms of time and money, incurred by medical tourists is C_4_. When medical institutions are successfully held accountable by medical tourists, the latter can receive compensation from medical institutions, which is recorded as C_0_, and C_0_ ≤ C_4_. When government departments actively regulate the medical tourism market to ensure its proper functioning, they may offer rewards to medical tourists who report privacy breaches by medical institutions. This is recorded as R.

*Hypothesis 5*: Government departments incur specific costs (C_5_) in the regulatory process of safeguarding privacy during the expansion of the medical tourism industry. These costs include formulating relevant policies and regulations, conducting publicity and supervision, as well as managing tourists’ accountable behaviors and social governance. Therefore, government departments will gain social credibility and be able to shape the image of destinations for medical tourism. Furthermore, they will gain economic and social benefits from the development of medical tourism, which will be recorded as B. These benefits include socio-economic growth, increased tax revenue generated by medical tourism, and the creation of employment opportunities. Choosing a “loose supervision” strategy means government departments will not bear the regulatory costs. However, the absence of regulation will result in an unfavorable social environment for the development of the medical tourism industry and leading to a decline in economic and social benefits, it is B′.

The relevant model parameters are set as shown in Table 1, and all parameters are non-negative.

**Table 1:** Meaning of parameter symbols and their representations.

Parameters	Indicates meaning
C_0_	Compensation to medical tourists under “negative protection” and “strict supervision” strategies.
C_1_	Costs to medical institutions under “active protection” strategy.
C_2_	Costs to medical institutions under “negative protection” strategy (C_2_ < C_1_).
I	Revenue from operations of medical institutions while providing services to medical tourists.
ΔI	Benefits gained by medical institutions under “negative protection.”
S	Subsidies to medical institutions under “active protection” and “strict supervision” strategies.
P_0_	Fines for medical institutions under “strict supervision” strategy (P_0_ ≤ ΔI).
L_1_	The image loss of medical institutions under “active protection” and “seek accountability” strategies.
L_2_	The image loss of medical institutions under “negative protection” and “strict supervision” strategies (L_1_ < L_2_).
C_3_	Costs for medical tourists when participating in medical tourism.
C_4_	Costs for medical tourists under “seek accountability” strategy (C_0_ < C_4_).
L_3_	Losses when medical tourists’ privacy is compromised.
V	The experiential value gained by medical tourists who participate in medical tourism.
R	Rewards for medical tourists under “seek accountability” and “strict supervision” strategies.
m	Probability of successful complaints by medical tourists under “loose supervision” strategy (0 < m < 1).
C_5_	Costs of government departments under “strict supervision” strategy.
B	Benefits gained by government departments under “strict supervision” strategy
B′	Benefits gained by government departments under “loose supervision” strategy
p	Probability of successful investigation and prosecution by government departments under “forgo accountability” (0 < p < 1)

### 3.3 Construction of gain function

**Figure 1:**
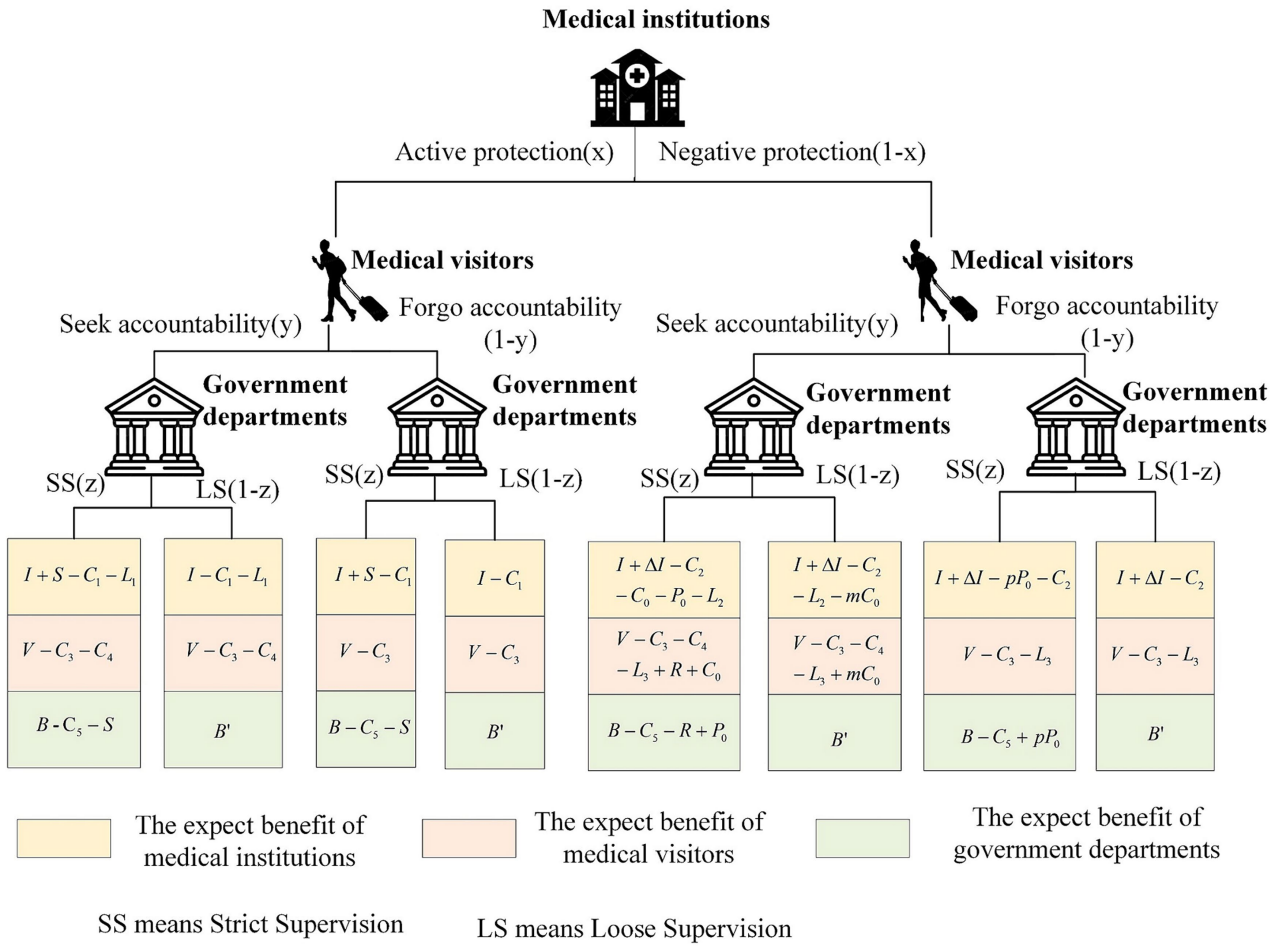
Benefits of each player.

The benefits of protecting the privacy of medical tourist participants are calculated based on the above assumption, as shown in Figure 1.

Based on the benefits, the payoffs for medical institutions, medical tourists, and government departments can be calculated.

For medical institutions, when choosing between the active protection strategy and the negative protection strategy, the expected benefits are U_11_ and U_12_, respectively. Therefore, the average expected benefit is U_1_.


$$\begin{array}{c} U_{11}=yz\left(I+S-C_{1}-L_{1}\right)+y\left(1-z\right)\left(I-C_{1}-L_{1}\right)\\ +z\left(1-y\right)\left(I+S-C_{1}\right)+\left(1-y\right)\left(1-z\right)\left(I-C_{1}\right) \end{array}$$

$$\begin{array}{c} U_{12}=yz\left(I+\Delta I-C_{2}-C_{0}-P_{0}-L_{2}\right)\\ +y\left(1-z\right)\left(I+\Delta I-C_{2}-L_{2}-mC_{0}\right)\\ +z\left(1-y\right)\left(I+\Delta I-pP_{0}-C_{2}\right)\\ +\left(1-y\right)\left(1-z\right)\left(I+\Delta I-C_{2}\right) \end{array}$$

$$U_{1}=xU_{11}+\left(1-x\right)U_{12}$$

For medical tourists, when they choose between “seek accountability strategy” and “forgo accountability” strategy, the expected benefits are U_21_ and U_22_, so the average expected benefit is U_2_.


$$\begin{array}{c} U_{21}=xz\left(V-C_{3}-C_{4}\right)+x\left(1-z\right)\left(V-C_{3}-C_{4}\right)\\ +z\left(1-x\right)\left(V-C_{3}-C_{4}-L_{3}+R+C_{0}\right)\\ +\left(1-x\right)\left(1-z\right)\left(V_{1}-C_{3}-C_{4}-L_{3}+mC_{0}\right) \end{array}$$

$$\begin{array}{c} U_{22}=xz\left(V-C_{3}\right)+x\left(1-z\right)\left(V-C_{3}\right)+z\left(1-x\right)\left(V-C_{3}-L_{3}\right)\\ +\left(1-x\right)\left(1-z\right)\left(V-C_{3}-L_{3}\right) \end{array}$$

$$U_{2}=yU_{21}+\left(1-y\right)U_{22}$$

For government departments, when choosing between strict supervision and loose supervision strategies, the expected benefits are U_31_ and U_32_, respectively. Therefore, the average expected benefit is U_3_.


$$\begin{array}{c} U_{31}=xy\left(B-C_{5}-S\right)+x\left(1-y\right)\left(B-C_{5}-S\right)\\ +y\left(1-x\right)\left(B-C_{5}-R+P_{0}\right)+\left(1-x\right)\left(1-y\right)\left(B-C_{5}+pP_{0}\right) \end{array}$$

$$U_{22}=xyB^{\prime }+x\left(1-y\right)B^{\prime }+y\left(1-x\right)B^{\prime }+\left(1-x\right)\left(1-y\right)B^{\prime }$$

$$U_{3}=zU_{31}+\left(1-z\right)U_{32}$$

### 3.4 Replicator dynamic equations for a three-party game

Based on the analysis of the expected benefits for the three parties involved in the game, the replicator dynamic equation can be derived. When medical institutions choose the active protection strategy, the replication dynamic equation is:


$$\begin{array}{c} F\left(x\right)=\frac{dx}{dt}=x\left(U_{11}-U_{1}\right)=x\left(1-x\right)\left(U_{11}-U_{12}\right)\\ =x\left(1-x\right)\left[\begin{array}{l} y\left(L_{2}-L_{1}+mC_{0}\right)+z\left(S+pP_{0}\right)\\ +yz\left(C_{0}+P_{0}-mC_{0}-pP_{0}\right)+C_{2}-C_{1}-\Delta I \end{array}\right] \end{array}$$

When medical tourists choose “seek accountability” strategy, the replication dynamic equation is:


$$\begin{array}{c} F\left(y\right)=\frac{\mathrm{dy}}{dt}=y\left(U_{21}-U_{2}\right)=y\left(1-y\right)\left(U_{21}-U_{22}\right)\\ =y\left(y-1\right)\left[\begin{array}{l} xmC_{0}-z\left(C_{0}+R-mC_{0}\right)\\ +xz\left(C_{0}+R-mC_{0}\right)-mC_{0} \end{array}\right] \end{array}$$

When government departments choose a strict supervision strategy, the replication dynamic equation is:


$$\begin{array}{c} F\left(z\right)=\frac{dz}{dt}=z\left(U_{31}-U_{3}\right)=z\left(1-z\right)\left(U_{31}-U_{32}\right)\\ =z\left(z-1\right)\left[\begin{array}{l} x\left(S+pP_{0}\right)+y\left(R-P_{0}+pP_{0}\right)\\ -xy\left(R-P_{0}+pP_{0}\right)+B^{\prime }-B+C_{5}-pP_{0} \end{array}\right] \end{array}$$

The three equations F(x), F(y), and F(z) are combined to create a replicator dynamic set of equations, namely:


$$\left\{\begin{array}{c} \begin{array}{c} F\left(x\right)=x\left(1-x\right)\left[\begin{array}{l} y\left(L_{2}-L_{1}+mC_{0}\right)+z\left(S+pP_{0}\right)\\ +yz\left(C_{0}+P_{0}-mC_{0}-pP_{0}\right)+C_{2}-C_{1}-\Delta I \end{array}\right]\\ F\left(y\right)=y\left(y-1\right)\left[\begin{array}{l} xmC_{0}-z\left(C_{0}+R-mC_{0}\right)\\ +xz\left(C_{0}+R-mC_{0}\right)-mC_{0} \end{array}\right]\; \end{array}\\ F\left(z\right)=z\left(z-1\right)\left[\begin{array}{l} x\left(S+pP_{0}\right)+y\left(R-P_{0}+pP_{0}\right)\\ -xy\left(R-P_{0}+pP_{0}\right)+B^{\prime }-B+C_{5}-pP_{0} \end{array}\right]\; \end{array}\right.$$

## 4 Model analysis

Based on the replicator dynamic equation system presented above, when F(x) = 0, F(y) = 0, and F(z) = 0, there are 8 partial equilibrium points that can be calculated as E_1_(0,0,0), E_2_(0,0,1), E_3_(0,1,0), E_4_(0,1,1), E_5_(1,0,0), E_6_(1,0,1), E_7_(1,1,0), E_8_(1,1,1). Among these points, E_1_(0,0,0) represents the equilibrium for the choices of “negative protection” by medical institutions, “forgo accountability” by medical tourists, and “loose supervision” by government departments. The other seven points have analogous interpretations. The asymptotically stable solution of the multi-group evolutionary game replicating dynamic system must be a strict Nash equilibrium solution, and it is a pure strategic Nash equilibrium (50). Thus, none of the state points are asymptotically stable, except for the eight local equilibria mentioned above, are asymptotically stable. Based on this, the stability of the evolutionary strategy of each participant in this game model is analyzed. Firstly, this study analyzes the asymptotic stability of medical institutions, medical tourists, and government departments. It then explores the evolutionary stability of the privacy protection system for medical tourists in the context of these measures.

### 4.1 Stability analysis of medical institutions

When analyzing the stability of medical institutions, the evolutionary stabilization strategy of medical institutions, denoted as x, is determined based on the replicator dynamic equation theorem, given F(x) = 0 and F′(x) < 0. Setting F(x) = 0, three solutions can be obtained: *x* = 0, *x* = 1, and y = [ΔI + C_1_ – C_2_ – z(S + pP_0_)] /[L_2_ – L_1_ + mC_0_ + z(C_0_ + P_0_ – mC_0_ – pP_0_)]. For ease of presentation, [ΔI + C_1_ – C_2_ – z(S + pP_0_)] /[L_2_ – L_1_ + mC_0_ + z(C_0_ + P_0_ – mC_0_ – pP_0_)] is simplified as ∏_1_. When y = ∏_1_, F(x) = 0, thus x takes any value in the interval is a steady state, and the probability of medical institutions’ strategy choice will not change over time. When y ≠ ∏_1_, consider the two strategies for medical institutions, where *x* = 0 and *x* = 1. The analysis regarding the stability of the group is divided into the following two scenarios: ① When 0 < y < ∏_1_, it can be judged that F′(0) < 0 and F′(1) > 0, therefore *x* = 0 is the evolutionary stabilization point. This indicates that when the probability that medical tourists choose to hold accountable is lower than ∏_1_, the medical institutions will choose a negative protection strategy. ② When ∏_1_ < y < 1, it can be judged that F′(0) > 0 and F′(1) < 0, therefore *x* = 1 is the evolutionary stabilization point. This indicates that when the probability that medical tourists choose to hold accountable is higher than ∏_1_, the medical institutions will choose an active protection strategy.

### 4.2 Stability analysis of medical tourists

When analyzing the stability of medical tourists, in accordance with the replicator dynamic equation theorem and considering situations F(y) = 0 and F′(y) < 0, the evolutionary stabilization strategy of medical tourists will be determined and denoted as y. Setting F(y) = 0, three solutions can be obtained: *y* = 0, *y* = 1, and *z* = (mC_0_ – mC_0_x – C_4_)/[(x – 1)(C_0_ + R – mC_0_)]. For ease of presentation, (mC_0_ – mC_0_x – C_4_)/[(x – 1)(C_0_ + R – mC_0_)] is simplified as ∏_2_. When z = ∏_2_ and F(y) = 0, y can take any value within the interval, resulting in a steady state, and the probability of medical tourists’ strategy choice will not change over time. For z ≠ ∏_2_, consider *y* = 0 and *y* = 1 as the two potential strategies for medical tourists. The analysis regarding the stability of the group is divided into the following two scenarios: ① When 0 < z < ∏_2_, it can be judged that F′(0) > 0 and F′(1) < 0, therefore, *y* = 1 is the evolutionary stabilization point. This suggests that if the probability of government departments opting for strict supervision is below ∏_2_, medical tourists will favor seeking accountability strategies. ② When ∏_2_ < z < 1, it can be judged that F′(0) < 0 and F′(1) > 0, therefore, *y* = 0 is the evolutionary stabilization point. This implies that if the probability of government departments opting for strict supervision exceeds ∏_2_, medical tourists will lean toward strategies forgoing accountability.

### 4.3 Stability analysis of government departments

When analyzing the stability of government departments, the evolutionary stabilization strategy of this group will be determined and denoted as z. This analysis is based on the replicator dynamic equation theorem and takes into consideration situation F(z) = 0 and F′(z) < 0. Setting F(z) = 0, three solutions can be obtained: *z* = 0, *z* = 1, and *x* = [B + pP_0_ – B′ – C_5_ – y(R – P_0_ + pP_0_)]/[S + pP_0_ – y(R – P_0_ + pP_0_)]. For ease of presentation, [B + pP_0_ – B′ – C_5_ – y(R – P_0_ + pP_0_)]/[S + pP_0_ – y(R – P_0_ + pP_0_)] is simplified as ∏_3_. When x = ∏_3_ and F(z) = 0, z can take any value within the interval, leading to a steady state, and the probability of government departments’ strategy choice will not change over time. When x ≠ ∏_3_, consider *z* = 0 and *z* = 1 as the two potential strategies for government departments. The analysis regarding the stability of the group is divided into the following two scenarios: ①When 0 < x < ∏_3_, it can be judged that F′(0) > 0 and F′(1) < 0, therefore *z* = 1 is the evolutionary stabilization point. This indicates that when the probability that medical institutions choose an active protection strategy is lower than ∏_3_, the government departments will choose strict supervision strategies. ②When ∏_3_ < x < 1, it can be judged that F′(0) < 0 and F′(1) > 0, therefore, *z* = 0 is the evolutionary stabilization points. This indicates that when the probability of medical institutions choosing an active protection strategy is higher than ∏_3_, government departments will choose loose supervision strategies.

### 4.4 Stability analysis of the decision-making system for privacy-protecting behaviors of medical tourists

The stability of the decision-making system concerning the privacy-protecting behaviors of medical tourists can be assessed by analyzing the eigenvalues of the Jacobian matrix. According to the first law of Lyapunov, if all the eigenvalues of the matrix are negative, the point is considered to be an asymptotically stable equilibrium point (51). Conversely, if there are positive eigenvalues, the point is considered unstable. The Jacobian matrix of the decision-making system for privacy protection behavior of medical tourists is as follows:


$$J_{\left(x\text{,}y\text{,}z\right)}=\left[\begin{array}{c} \frac{\partial F\left(x\right)}{\partial x}\frac{\partial F\left(x\right)}{\partial y}\frac{\partial F\left(x\right)}{\partial z}\\ \frac{\partial F\left(y\right)}{\partial x}\frac{\partial F\left(y\right)}{\partial y}\frac{\partial F\left(y\right)}{\partial z}\\ \frac{\partial F\left(z\right)}{\partial x}\frac{\partial F\left(z\right)}{\partial y}\frac{\partial F\left(z\right)}{\partial z} \end{array}\right]=\left[\begin{array}{c} F_{11}\;F_{12}\;F_{13}\\ F_{21}\;F_{22}\;F_{23}\\ F_{31}\;F_{32}\;F_{33} \end{array}\right]$$

In the above formula:


$$F_{11}=\left(1-2x\right)\left[\begin{array}{l} y\left(L_{2}-L_{1}+mC_{0}\right)+z\left(S+pP_{0}\right)\\ +yz\left(C_{0}+P_{0}-mC_{0}-pP_{0}\right)+C_{2}-C_{1}-\Delta I \end{array}\right]$$

$$F_{12}=x\left(1-x\right)\left[L_{2}-L_{1}+mC_{0}+z\left(C_{0}+P_{0}-mC_{0}-pP_{0}\right)\right]$$

$$F_{13}=x\left(1-x\right)\left[S+pP_{0}+y\left(C_{0}+P_{0}-mC_{0}-pP_{0}\right)\right]$$

$$F_{21}=y\left(y-1\right)\left[mC_{0}+z\left(C_{0}+R-mC_{0}\right)\right]$$

$$F_{22}=\left(2y-1\right)\left[\begin{array}{l} xmC_{0}-z\left(C_{0}+R-mC_{0}\right)\\ +xz\left(C_{0}+R-mC_{0}\right)+C_{4}-mC_{0} \end{array}\right]$$

$$F_{23}=y\left(y-1\right)\left[-\left(C_{0}+R-mC_{0}\right)+x\left(C_{0}+R-mC_{0}\right)\right]$$

$$F_{31}=z\left(z-1\right)\left[S+pP_{0}-y\left(R-P_{0}+pP_{0}\right)\right]$$

$$F_{32}=z\left(z-1\right)\left[R-P_{0}+pP_{0}-x\left(R-P_{0}+pP_{0}\right)\right]$$

$$F_{33}=\left(2z-1\right)\left[\begin{array}{l} x\left(S+pP_{0}\right)+y\left(R-P_{0}+pP_{0}\right)\\ -xy\left(R-P_{0}+pP_{0}\right)+B^{\prime }-B+C_{5}-pP_{0} \end{array}\right]$$

By incorporating each of the eight equalization points E_1_(0,0,0), E_2_(0,0,1), E_3_(0,1,0), E_4_(0,1,1), E_5_(1,0,0), E_6_(1,0,1), E_7_(1,1,0), and E_8_(1,1,1) into the Jacobian matrix, the corresponding eigenvalues will be obtained, as presented in Table 2.

**Table 2:** Eigenvalues of the Jacobian matrix.

Balance points	Eigenvalue λ_1_	Eigenvalue λ_2_	Eigenvalue λ_3_
E_1_(0,0,0)	C_2_ – C_1_ – ΔI	–(C_4_ – mC_0_)	–(B′ – B + C_5_ -pP_0_)
E_2_(0,0,1)	–(C_2_ – C_1_ – ΔI)	–C_4_	–(S + B′ – B + C_5_)
E_3_(0,1,0)	L_2_ – L_2_ + mC_0_ + C_2_ – C_1_ – ΔI	C_4_ – mC_0_	–(R – P_0_ + B′ – B + C_5_)
E_4_(0,1,1)	S + pP_0_ + C_2_ – C_1_ – ΔI	–(C_4_ – C_0_ – R)	B′ – B + C_5_ -pP_0_
E_5_(1,0,0)	–(L_2_ – L_2_ + mC_0_ + C_2_ – C_1_ – ΔI)	C_4_	–(S + B′ – B + C_5_)
E_6_(1,0,1)	–(S + pP_0_ + C_2_ – C_1_ – ΔI)	–C_4_	S + B′ – B + C_5_
E_7_(1,1,0)	L_2_ – L_1_ + S + C_0_ + P_0_ + C_2_ – C_1_ – ΔI	C_4_ – C_0_ – R	R – P_0_ + B′ – B + C_5_
E_8_(1,1,1)	–(L_2_ – L_1_ + S + C_0_ + P_0_ + C_2_ – C_1_ – ΔI)	C_4_	S + B′ – B + C_5_

In a real-world scenario, the initial parameters should satisfy the conditions R + C_0_ – C_4_ > 0 and B – C_5_ – S – B_2_ > 0. This implies that medical tourists receive greater compensation from medical institutions and rewards from government departments in the event of successful litigation than the costs of their legal proceedings. Additionally, the economic and social benefits of strict supervision by government departments exceed the gains from lax supervision. Based on previous assumptions, namely C_1_ > C_2_, L_1_ < L_2_, B > B′, and C_0_ < C_4_, the equilibrium points E_1_(0,0,0), E_2_(0,0,1), E_3_(0,1,0), E_4_(0,1,1), E_5_(1,0,0), and E_8_(1,1,1) do not satisfy the conditions necessary for determining the sign of the eigenvalue of a stable point. The eigenvalue sign determinations for E_6_(1,0,1) and E_7_(1,1,0) warrant further discussion. Based on the provided conditions, the stability analysis for each equilibrium point can be found in Table 3.

**Table 3:** Stability analysis of equilibrium points.

Balance points	Eigenvalue λ_1_	Eigenvalue λ_2_	Eigenvalue λ_3_	Stability conditions
E_1_(0,0,0)	−	−	+	Unstable point
E_2_(0,0,1)	+	−	+	Unstable point
E_3_(0,1,0)	±	+	±	Unstable point
E_4_(0,1,1)	±	+	−	Unstable point
E_5_(1,0,0)	±	+	+	Unstable point
E_6_(1,0,1)	±	−	−	When S – C_1_ > ΔI – C_2_ -pP_0_, it’s an evolutionary stabilization strategy.
E_7_(1,1,0)	±	−	±	When S – L_1_ – C_1_ < ΔI –C_2_ – L_2_ – C_0_ – P_0_ and B- C_5_ – R + P_0_ > B′, it’s an evolutionary stabilization strategy.
E_8_(1,1,1)	±	+	−	Unstable point

As can be seen from Table 3, the evolutionary game equilibrium of the behavioral strategies of subjects involved in protecting the privacy of medical tourists is influenced by various factors. The detailed analysis of the two evolutionary stabilization strategies E_6_(1,0,1) and E_7_(1,1,0) are as follows:

When S – C_1_ > ΔI – C_2_ – pP_0_ and B′ < B – C_5_ – S, E_6_(1,0,1) — represented as (active protection, forgo accountability, strict supervision) — emerges as an evolutionary stabilization strategy. Under these conditions: ① The difference between the government subsidy and the costs borne by medical institutions for actively protecting the privacy of medical tourists exceeds the gap between the profit they earn from breaching this privacy and the combined expenses and fines levied by government departments. Given this, medical institutions have a stronger inclination to prioritize the protection of medical tourists’ privacy. ② In situations where medical institutions proactively ensure privacy and face strict government regulations, medical tourists adopting seeking accountability strategy end up shouldering the related costs without any accompanying compensation. Consequently, the “forgo accountability” option is more appealing for medical tourists. ③ When the overall benefits obtained by government departments from actively supervising the privacy protection behaviors of medical institutions exceed the costs of active supervision and the subsidies for the institutions’ proactive protection actions, and are greater than the economic and social benefits from passive supervision, the government departments, based on the principle of maximizing benefits, have sufficient motivation to maintain the order of the medical tourism market development and to actively oversee the privacy protection measures of medical institutions.

When S – L_1_ – C_1_ < ΔI – C_2_ – L_2_ – C_0_ -P_0_, C_4_ < C_0_ + R and B – C_5_ – R + P_0_ > B′, E_7_(1,1,0), representing the (negative protection, seek accountability, strict supervision) strategy, emerges as an evolutionary stabilization strategy. The primary reason behind this outcome is the disparity between the profits gained and potential repercussions for medical institutions that compromise the privacy of medical tourists. This includes costs arising from breaches of privacy, reputation damage, potential compensation to affected tourists, and fines imposed by government departments. This disparity outweighs the difference between government subsidies, the cost of proactive protection, and the reputational damage to medical tourists when they are erroneously held accountable. Consequently, medical institutions frequently choose negative protection measures to prioritize their interests. When medical tourists experience privacy breaches and decide to hold medical institutions accountable, the total compensation they receive, combined with the government’s rewards, surpasses the cost of seeking accountability. In this scenario, holding medical institutions responsible is the most favorable strategy for medical tourists. Regarding government departments, they stringently monitor the malpractices of medical institutions. Even though they shoulder the costs of regulation and incentives for medical tourists, they also levy fines on medical institutions for any breaches. Nevertheless, the net benefit (B – C_5_ – R + P_0_) still surpasses the economic and social benefits B′ derived from the government’s lenient supervision approach. Thus, the government department leans toward a “strict supervision” strategy.

Comparing the two evolutionary stabilization strategies mentioned above and considering the processes of collecting, utilizing, and safeguarding the privacy of medical tourists, the optimal strategy for evolutionary stabilization should be E_6_(1,0,1). In other words, the optimal strategy is achieved when medical institutions choose “active protection,” medical tourists opt for “forgo accountability,” and government departments select “strict supervision.” In this case, medical institutions demonstrate a strong subjective initiative in the process of collecting, storing, sharing, and analyzing the private information of medical tourists during the provision of medical services. Under the strict supervision of government departments, they continuously optimize privacy protection technologies and systems to help prevent unauthorized access, tampering, and disclosure of medical tourists’ personal information. This fortifies the foundation for the sustainable development of the medical tourism industry. Beyond experiential value, the effective protection of personal information has become the primary concern for medical tourists. The collaboration between medical institutions and government departments in fostering a robust medical tourism market and enhancing the healthcare environment can diminish privacy and security worries for medical tourists. In turn, this encourages tourists to actively and willingly engage in medical tourism activities. As an emerging industry, the medical tourism sector is pivotal in stimulating domestic demand, stabilizing economic growth, creating job opportunities, elevating people’s quality of life, and ensuring the health of residents. It stands as one of the future industries in healthcare poised to boost regional competitiveness.

## 5 Simulation analysis

To more clearly and intuitively depict the dynamic evolution process of strategy choices among medical institutions, medical tourists, and government departments, the Matlab R2016b software is employed to numerically simulate the established three-party evolution game model. The primary goal is to compare the effects of various participants on the protection of medical tourists’ privacy under a system of incentives and penalties. This study aims to understand the principles and regulations that govern the impact of government departments’ regulatory methods and efforts on the effectiveness of privacy protection for medical tourists, especially when there’s limited supervision cost. Specifically, the study examines how punitive measures imposed by government departments on medical institutions, the compensation provided by these institutions to medical tourists, the subsidies from government departments to medical institutions, and the rewards given to medical tourists for lodging valid complaints influence the dynamics of this evolutionary game.

### 5.1 The effect of reward and punishment factors on the evolutionary path of participating subjects in the scenario E_6_(1,0,1)

In light of the current situation and the stability analysis conditions of E_6_(1,0,1), the initial values of the parameters used in this study are provided in Table 4. For the behavioral strategy choices in the three-party evolutionary game, the initial probabilities of strategy choices for medical institutions, medical tourists, and government departments are set to (*x* = 0.5, *y* = 0.5, *z* = 0.5). This setup allows for simulation under various reward and punishment intensities.

**Table 4:** Initial value of the parameter.

Parameters	C_1_	C_2_	C_4_	C_5_	L_1_	L_2_	I	ΔI	S	R	B	B’	C_0_	P_0_	p	m
Initial value	20	10	5	25	8	20	25	20	35	10	90	20	5	20	0.1	0.05

#### 5.1.1 The effect of different initial strategy selection probabilities on the evolutionary path of participating subjects

**Figure 2:**
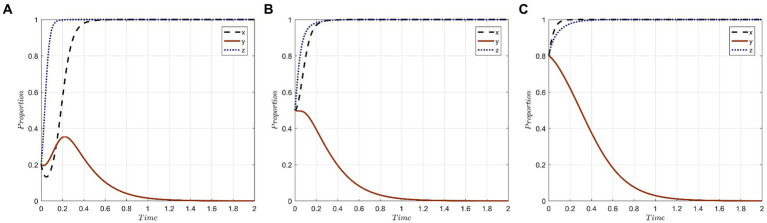
Evolutionary trajectories of the system under different initial probabilities of x,y,z: **(A)** The initial probability is 0.2; **(B)** The initial probability is 0.5; **(C)** The initial probability is 0.8.

This study assumes that the initial probability for a medical institution to choose the “active protection” strategy is x_0_, for medical tourists to select the “seek accountability” strategy is y_0_, and for government departments to opt for the “strict supervision” strategy is z_0_. The combined initial probability for the tripartite evolutionary game system is represented as (x_0_, y_0_, z_0_). We consider three sets of probability values: (0.2, 0.2, 0.2), (0.5, 0.5, 0.5), and (0.8, 0.8, 0.8). The evolutionary trajectories based on these initial probabilities are illustrated in Figure 2.

The key observations from Figure 2 are as follows: Medical institutions, when leaning toward the “active protection” strategy from the onset, exhibit a faster evolutionary speed, which reduces the time required to achieve strategy stability. Their decision-making is swift and independent, often tailored to their specific circumstances and benefits. Across all initial probabilities, medical tourists predominantly opt for the “forgo accountability” strategy. With an initial probability of 0.2, there’s a preference for the “seek accountability” strategy, but this gradually shifts to “forgo accountability.” The most rapid stabilization is observed when the initial probability is 0.5. This suggests that medical tourists’ behaviors are influenced by public sentiment and are characterized by a pattern of learning and conformity. Regardless of variations in initial probability, the time taken for government departments to stabilize their strategy remains consistent. This indicates a characteristic stability in their strategic evolution.

#### 5.1.2 The effect of the government department’s punishment of medical institutions on the behavior of the three-party evolutionary game

**Figure 3:**
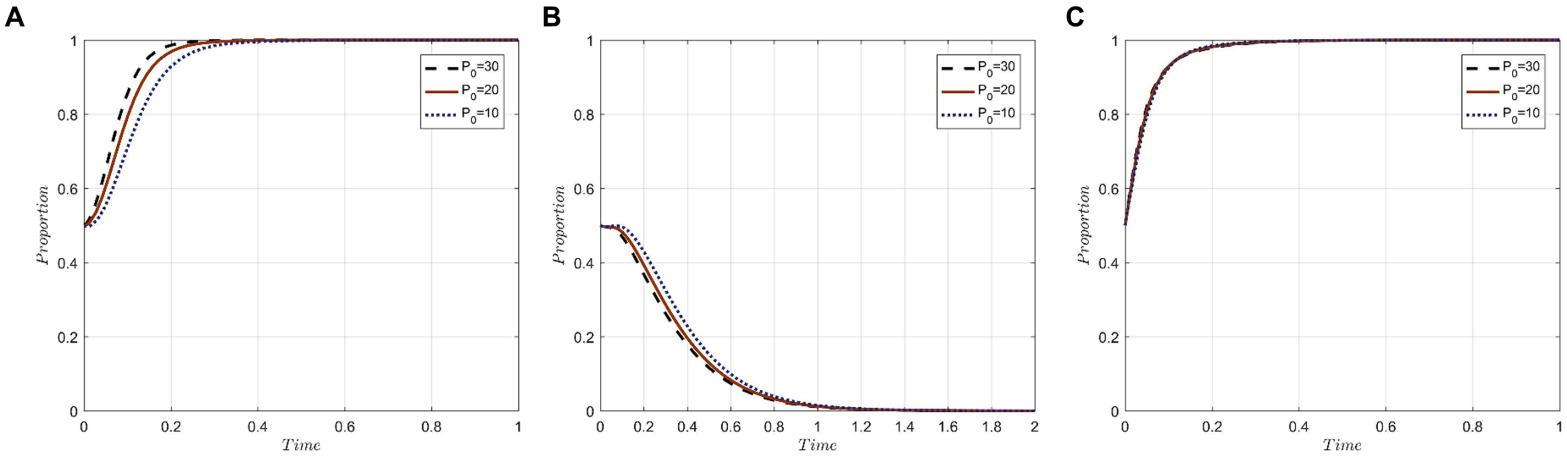
Evolutionary trajectory of tripartite behavior under different punishment strengths: **(A)** Evolutionary trajectories of x; **(B)** Evolutionary trajectories of y; **(C)** Evolutionary trajectories of z.

In order to represent varying degrees of punishment, it is assumed that P_0_ takes the values of 30, 20, and 10, symbolizing high, medium, and low punishment levels, respectively. Figure 3 depicts the simulation results of the three parties under these different punishment strengths.

The results indicate that escalating penalties imposed by the government can expedite the attainment of the evolutionary stable state in the three-party evolutionary game system. For medical institutions, a rise in government fines directly increases the cost of neglectfully protecting the privacy of medical tourists. Such fines deter opportunistic behaviors by medical institutions aiming to benefit at the expense of the privacy of medical tourists, thus steering the strategy evolution toward “active protection.”

For medical tourists, irrespective of the punishment’s severity, they consistently lean toward “forgo accountability” strategies. Notably, evolution is swifter with stiffer penalties. In other words, the more stringent the fines imposed by government departments for privacy breaches, the more inclined medical tourists are to opt for a “forgo accountability” strategy. This inclination stems from medical tourists’ heightened trust in robust government regulation underpinned by stringent penalties. With this trust, they believe their privacy will be effectively upheld, rendering them less likely to hold medical institutions accountable.

For government departments, the three penalty tiers exhibit minimal influence over the likelihood of them selecting the “strict supervision” strategy. Nonetheless, there’s a discernible acceleration in evolution as penalty levels escalate. The reason being, under the purview of government departments with stringent penalties, medical institutions more actively safeguard the privacy of medical tourists, thereby amplifying societal benefits. This enhancement aligns with the primary objective of government departments’ proactive regulation. Hence, punitive measures emerge as a pivotal regulatory instrument for government departments in ensuring the seamless functioning of the medical tourism market.

#### 5.1.3 The effect of medical institutions’ compensation efforts for medical tourists on the behavior of the three-party evolutionary game

**Figure 4:**
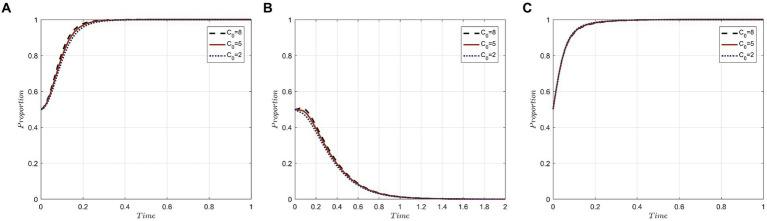
Evolutionary trajectory of tripartite behavior under different levels of compensation: **(A)** Evolutionary trajectories of x; **(B)** Evolutionary trajectories of y; **(C)** Evolutionary trajectories of z.

To investigate the influence of compensation levels offered by medical institutions on the evolutionary trajectory of the tripartite behaviors, we assign values of 8, 5, and 2 to C_0_, representing high, medium, and low compensation strengths, respectively. The simulation results under these different compensation levels are presented in Figure 4.

The results indicate that compensation levels significantly impact the strategic choices of both medical institutions and medical tourists. However, the government sector remains relatively unaffected by these variations. The rationale behind this lies in the core objectives of each party.

For medical institutions, their primary goal is revenue generation and profit maximization through the provision of medical tourism services. Although employing a negative protection strategy could lead to cost savings and potential profits from unauthorized privacy disclosures, the costs associated with violations increase with the magnitude of the required compensation when breaches are identified and penalized. Thus, facing higher compensation demands makes medical institutions more inclined toward the “active protection” strategy.

For medical tourists, their participation in medical tourism aims at physical rejuvenation, mental well-being, and overall satisfaction. When their privacy gets compromised, it tarnishes their perception of both the individual medical institutions and the broader medical tourism industry. In scenarios of higher compensation, medical tourists are increasingly motivated to hold the institutions accountable, both to protect their rights and to mitigate potential financial setbacks.

For government departments, their regulatory interventions precede the compensation actions by medical institutions. Consequently, the degree of compensation has a negligible influence on the strategic decisions of government entities.

#### 5.1.4 The effect of government departments’ subsidy strength to medical institutions on the behavior of the three-party evolutionary game

**Figure 5:**
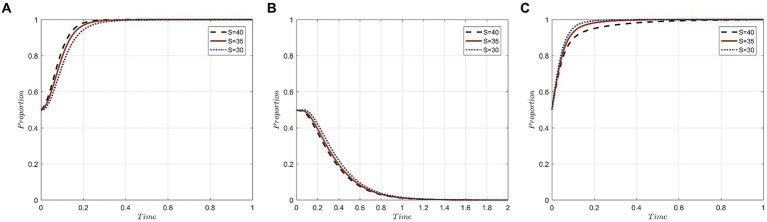
Evolutionary trajectories of tripartite behavior under different subsidy strengths: **(A)** Evolutionary trajectories of x; **(B)** Evolutionary trajectories of y; **(C)** Evolutionary trajectories of z.

We assume values of 40, 35, and 30 for S, representing different levels of subsidies provided by government departments to medical institutions. The corresponding simulation results are depicted in Figure 5.

In the realm of medical institutions and medical tourists, the subsidy level offered by government departments serves as a direct indicator of governmental support for the growth of the medical tourism industry. This subsidy level also symbolizes a conducive market environment for the industry. Within such a setting, medical institutions tend to favor an “active protection” strategy, whereas medical tourists lean toward a “forgo accountability” approach. This dynamic facilitates value creation through collaboration among various stakeholders in the medical tourism sector.

As for government departments, a preference for “strict supervision” is observed across different subsidy levels. However, it’s notable that when offering substantial subsidies to medical institutions, it takes a longer duration for these government departments to achieve strategic stability. Consequently, the magnitude of subsidy expenditure emerges as a pivotal factor influencing the strategic decisions of government departments.

#### 5.1.5 The effects of government department incentives for medical tourists on the behavior of the three-way evolutionary game

**Figure 6:**
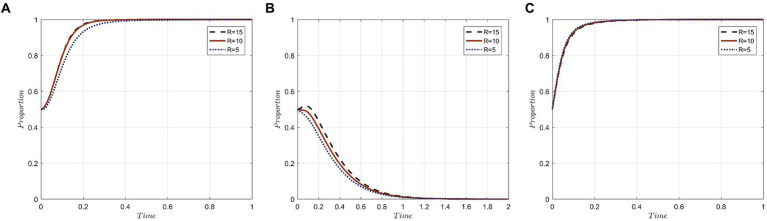
Evolutionary trajectory of tripartite behavior under different reward strengths: **(A)** Evolutionary trajectories of x; **(B)** Evolutionary trajectories of y; **(C)** Evolutionary trajectories of z.

Values of 15, 10, and 5 are assigned to R, representing the extent to which government departments reward medical tourists for holding medical institutions accountable when their privacy is breached. The simulation results are depicted in Figure 6. As observed in the figure, the strength of incentives influences the strategic choices of medical institutions. With medium and high reward strengths, medical institutions achieve an evolutionary stable state more swiftly. However, the impact of an exceedingly high reward strength on the strategic choices of medical institutions plateaus, with medium reward strength exerting the most influence. It suggests that moderate public scrutiny boosts the likelihood of medical institutions actively safeguarding medical tourists’ privacy. For medical tourists, there’s an initial inclination toward being swayed by regulatory incentives from government departments, advocating for active privacy protection by medical institutions. Over time, however, “forgo accountability” emerges as the predominant preference among medical tourists. Concurrently, the time required for medical institutions to achieve stability extends, pointing to an inverse relationship between evolutionary rate and incentive strength. To ensure a secure and structured medical tourism environment, government departments proactively regulate the market by incentivizing medical tourists to uphold responsible behaviors. The strict supervision approach of government departments remains relatively constant, regardless of incentive variations, underscoring their commitment to ensuring positive experiences for medical tourists. This promotes a healthy medical tourism industry, where the privacy of medical tourists is paramount for medical institutions.

### 5.2 The analysis of evolution path from E_7_(1,1,0) to E_6_(1,0,1)

**Figure 7:**
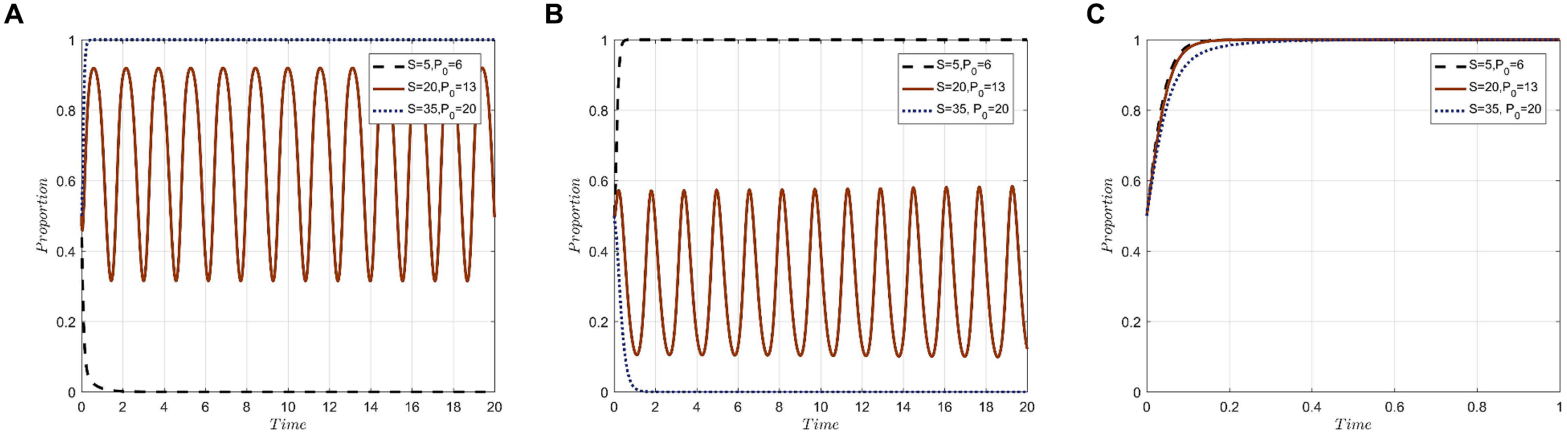
Evolution results of simultaneous changes of S and P_0_ under condition E_7_(0,1,1): **(A)** Evolutionary trajectories of x; **(B)** Evolutionary trajectories of y; **(C)** Evolutionary trajectories of z.

Given the initial parameters, S and P_0_ are set to values of 5 and 6, respectively. These values ensure the parameter combinations meet the conditions for the evolutionary stabilization strategy E_7_(1,1,0): S – L_1_ – C_1_ < ΔI – C_2_ – L_2_ – C_0_ – P_0_, C_4_ < C_0_ + R, while B – C_5_ – R + P_0_ > B′. For the pairs (S, P_0_), values (5, 6), (20, 13), and (35, 20) are assigned to simulate scenarios where government departments amplify regulations alongside increasing subsidies and penalties. Starting from an initial state of (0.5, 0.5, 0.5), the outcomes are displayed in Figure 7. The evolutionary results pinpoint two transitions in the steady state of the tripartite game system: first, from E_7_(1,1,0) to an unstable state with no stabilization strategy, and second, from the unstable state to strategy E_6_(1,0,1). The end result highlights the “active protection” by medical institutions, “forgo accountability” by medical tourists, and “strict supervision” by government departments. This can be attributed to heightened regulatory efforts by government departments, which leads to compliant medical institutions gaining more in subsidies, while non-compliant ones face stiffer penalties. In an ecosystem balanced with ample incentives and strict punitive measures, medical institutions lean more toward safeguarding medical tourists’ privacy. When governmental oversight is lax, medical institutions might lean toward a “negative protection” strategy, reducing trust among medical tourists and prompting them to lean into “seek accountability” strategy. However, as governmental regulations tighten, marked by increased subsidies and penalties, the trust in medical institutions grows among medical tourists. Consequently, the strategy of medical tourists gradually shifts to “forgo accountability.”

## 6 Conclusion and limitations

### 6.1 Conclusion

Through an evolutionary game theoretic lens, two primary stable strategies have emerged: E_6_(1,0,1) and E_7_(1,1,0). Of these, E_6_(1,0,1) is identified as the pinnacle strategy for assuring medical tourists’ privacy. This optimal strategy is a confluence of the “active protection” by medical institutions, “forgo accountability” taken up by medical tourists, and the “strict supervision” by government departments. The stability of this evolutionary strategy is predominantly swayed by factors such as penalties from government bodies for lax privacy protection, compensations from institutions to tourists, governmental aids to institutions, and accolades to tourists who rightly pinpoint institutions for privacy lapses.

Simulations based on the preferred stabilization strategy, E_6_(1,0,1), reveal the following: decision-making by medical institutions is swift and autonomous; medical tourists’ decisions are influenced by learning and societal norms; and government actions remain consistent. Enhancing incentives and penalties bolsters institutions’ efforts to actively protect the privacy of medical tourists. Furthermore, when medical tourists receive significant incentives, either through compensation from institutions or rewards from the government, there is a noticeable extension in the time required to converge to the “forgo accountability” evolutionary stabilization approach. This highlights the critical influence of tourists’ vested interests. An increase in governmental subsidies to medical institutions might deter their willingness for “strict supervision.” Exorbitantly high subsidy amounts increase the government’s regulatory costs, resulting in a more lenient supervision approach in the medical tourism market.

A recalibration of incentives and penalties by government bodies effectively encourages institutions to adopt “active protection,” guides tourists toward “forgo accountability,” and reinforces “strict supervision” by government departments. This recalibration facilitates a transition from strategy E_7_(1,1,0) to the optimal strategy E_6_(1,0,1), promoting sustainable medical tourism.

### 6.2 Implications

This study articulates strategies for a sustainable medical tourism industry, emphasizing regulatory cost management and a rational incentive system based on tripartite evolutionary game theory simulations. Government bodies are encouraged to optimize subsidies and penalties, focusing on merit-based rewards for medical institutions that excel in privacy protection. This approach aims to balance regulatory efficiency with fiscal prudence, promoting comprehensive privacy safeguards. Moderate penalties are recommended to avoid covert privacy breaches, while interdepartmental collaboration is highlighted as crucial for unified governance. The integration of advanced technologies and the establishment of an information disclosure platform are identified as key measures to enhance transparency and facilitate dual oversight. These strategies advocate for a sustainable medical tourism framework that prioritizes privacy protection, technological innovation, and transparent governance, ultimately improving the medical tourism experience by safeguarding patient privacy and enhancing service efficiency.

In light of the findings from an evolutionary game theoretic analysis of medical tourism, this study proposes several practical implications for academic scholars and organizations within the field. The identification of E_6_(1,0,1) as the optimal strategy for ensuring the privacy of medical tourists underscores the need for a multifaceted approach, encompassing active protection by medical institutions, a redefined sense of accountability among tourists, and stringent government supervision. To translate these insights into practice, it is recommended that scholars engage in further research to refine models simulating the effects of various incentive structures, thereby enriching the empirical basis for policy adjustments. Concurrently, organizations should leverage these insights to advocate for policy reforms that align with the optimal strategy, emphasizing the balance of incentives and penalties to encourage privacy protection without compromising the viability of the medical tourism sector. Additionally, educational initiatives aimed at stakeholders across the medical tourism spectrum can enhance understanding and implementation of best practices in privacy protection. The development of technological solutions, facilitated by collaborations between academics, technology firms, and medical tourism practitioners, can further support the enactment of the identified strategy. Finally, cross-sector collaboration is essential for fostering a unified approach to privacy protection, ensuring that the medical tourism industry advances in a manner that is sustainable, ethical, and respectful of privacy rights. Through these concerted efforts, the theoretical insights derived from evolutionary game theory can be effectively applied to address the complex dynamics of privacy protection in medical tourism, contributing to the field’s ongoing development and the establishment of robust privacy safeguards.

### 6.3 Limitations

This study, centered on the evolutionary game model’s insights into the privacy protection behaviors of medical tourists, exhibits certain limitations. Primarily, the model may not encapsulate the entire spectrum of real-world intricacies inherent in the interactions among the principal actors: medical tourists, institutions, and government entities. The research somewhat confines itself to predetermined strategic sets, with a predominant focus on economic drivers, potentially sidelining psychological or cultural nuances. Furthermore, the primary emphasis on strategies E_6_(1,0,1) and E_7_(1,1,0) might restrict its universal applicability across diverse healthcare landscapes. Future research avenues could delve deeper into introducing varied strategic combinations, understanding non-economic determinants of behavior, and appraising the model’s validity across different healthcare systems. Incorporation of technological impacts on privacy strategies, given the ascent of digital health paradigms, remains a promising domain for subsequent inquiries.

## Data availability statement

The original contributions presented in the study are included in the article/supplementary material, further inquiries can be directed to the corresponding author.

## Author contributions

RW: Conceptualization, Data curation, Formal analysis, Investigation, Methodology, Resources, Software, Validation, Visualization, Writing – original draft, Writing – review & editing. SG: Supervision, Writing – review & editing.
